# Effects of aging on the histology and biochemistry of rat tendon healing

**DOI:** 10.1186/s12891-021-04838-w

**Published:** 2021-11-15

**Authors:** Fan Lai, Hong Tang, Jingjing Wang, Kang Lu, Xuting Bian, Yunjiao Wang, Youxing Shi, Yupeng Guo, Gang He, Mei Zhou, Xuan Zhang, Binghua Zhou, Jiqiang Zhang, Wan Chen, Kanglai Tang

**Affiliations:** 1 Department of Sports Medicine Center, Southwest Hospital, Army Medical University, Chongqing, 400038 China; 2 Department of Blood Transfusion, Xinqiao Hospital, Army Medical University, 400037 Chongqing, China; 3 Department of Neurobiology, Army Medical University, 400038 Chongqing, China

**Keywords:** Tendon healing, Aging, Collagen synthesis, Adipocyte accumulation, Ectopic ossification

## Abstract

**Introduction:**

Tendon diseases and injuries are a serious problem for the aged population, often leading to pain, disability and a significant decline in quality of life. The purpose of this study was to determine the influence of aging on biochemistry and histology during tendon healing and to provide a new strategy for improving tendon healing.

**Method:**

A total of 24 Sprague-Dawley rats were equally divided into a young and an aged group. A rat patellar tendon defect model was used in this study. Tendon samples were collected at weeks 2 and 4, and hematoxylin-eosin, alcian blue and immunofluorescence staining were performed for histological analysis. Meanwhile, reverse transcription-polymerase chain reaction (RT-PCR) and western blot were performed to evaluate the biochemical changes.

**Results:**

The histological scores in aged rats were significantly lower than those in young rats. At the protein level, collagen synthesis-related markers Col-3, Matrix metalloproteinase-1 and Metallopeptidase Inhibitor 1(TIMP-1) were decreased at week 4 in aged rats compared with those of young rats. Though there was a decrease in the expression of the chondrogenic marker aggrecan at the protein level in aged tendon, the Micro-CT results from weeks 4 samples showed no significant difference(*p*>0.05) on the ectopic ossification between groups. Moreover, we found more adipocytes accumulated in the aged tendon defect with the Oil Red O staining and at the gene and protein levels the markers related to adipogenic differentiation.

**Conclusions:**

Our findings indicate that tendon healing is impaired in aged rats and is characterized by a significantly lower histological score, decreased collagen synthesis and more adipocyte accumulation in patellar tendon after repair.

**Supplementary Information:**

The online version contains supplementary material available at 10.1186/s12891-021-04838-w.

## Introduction

Musculoskeletal disorders and injuries are a considerable problem for the aged population, resulting in pain, disability, and substantial declines in quality of life. Tendon injuries are no exception; it has been reported that the incidence of rotator cuff tears is greater than 25% in people over the age of 60 [1]. Aging is often viewed as an important risk factor for tendon disorders [2], though a recent paper by Gagliano et al. demonstrated that the structure and composition of ECM in ageing tendons are preserved as well as the expression of genes and proteins involved in collagen turnover pathways [3], other studies showed that the healing effect of tendon injuries in aged patients was often impaired [4–6]. However, basic research studies have not evaluated the influence of aging on tendon healing after tendon injury.

It is difficult to restore the normal structure and function of injured tendons by existing medical and surgical treatments [7]. Most of the treatments for tendon injuries are based on current limited clinical experience, rather than knowledge about the pathogenic alterations in the process of tendon healing in aged individuals. In fact, therapies used in the treatment of age-related tendon diseases are often accompanied by negative complications, such as re-tear of the rotator cuff after surgical treatments in older patients [8, 9]. Therefore, it is necessary to explore the pathological changes in the healing of aged tendons and find therapeutic targets for the management of age-related tendon disabilities.

It has been reported that collagen synthesis in tendons reduces drastically with advancing age [10], even though the diameter of collagen fibrils appears to remain unchanged [11]. Key genes associated with extra cellular matrix (ECM) remodeling were found to be downregulated in the tendons of old rats [12]. Vinicius revealed that in comparison to their levels in young animals, collagen-I (Col-I) content and glucosaminoglycans (GAGs) were reduced in the calcaneal tendon of older sedentary animals, while biglycan (Bgn) and fibromodulin (Fmod) content were not altered with aging. The gene expression levels of COL-1, COL-3, Insulin-Like Growth Factor-Ia (IGF-Ia), Matrix metalloproteinase-2(MMP-2), tissue inhibitor of metalloproteinase-2(TIMP-2), and Bgn were decreased in aged tendons [13].

In this study, a rat acute patellar tendon injury model was established, and the biochemistry and histology of the healing tendons were determined. This animal model was widely used in the researches of tendon healing or regeneration, especially in the field of aging [14, 15]. The results showed that aged rat tendons had inferior gross appearance. In addition, aging may have impaired the collagen synthesis by reducing the expression levels of MMPs and TIMPs and affected the differentiation of TSPCs in tendons. These cues may provide us with new strategies for treating age-related tendon injuries.

## Materials and methods

### Ethics statement

Two-month-old male Sprague Dawley rats (200–250 g) and 20-month-old rats (550–650 g) were used as young and aged rats, respectively. All rats had free access to water and food and they were raised under a light-dark cycle of 12 h and with a controlled room temperature (23–25 °C). All procedures were conducted in strict accordance with the institutional guidelines for laboratory animal treatment and care and were approved by the Army Medical University of Institutional Animal Care and Use Committee (IACUC).

### Animal model

A total of 30 Sprague Dawley rats were equally divided into 2 groups: a young group and an aged group. To create a tendon defect, the central portion of the patellar tendon (1 mm in width) from the distal apex of the patella to the insertion of the tibia tuberosity was removed without damaging the fibrocartilage zone; the procedure was performed with two stacked sharp blades as described in a previous study [16]. The tendon defects were created on both sides. At weeks 2 and 4, 6 rats in each group were sacrificed by CO_2_ asphyxiation with a flow rate of 30% of chamber volume per minute, the whole tendon was detached and all the patellar tendons were collected for histological and biochemical analyses. Three rats (6 tendons) in each group were used for microCT scanning at weeks 8 post injury.

### Hematoxylin and eosin *(HE), Alcian blue and oil red O staining*

The patellar tendons were fixed in buffered formalin for 24 h, and tendons were dehydrated and embedded in optimal cutting temperature compound (OCT); then, the tissue was cut to produce longitudinal sections (5 μm) as previously described [17]. After washing three times with phosphate buffered saline (PBS), the sections were stained with hematoxylin and eosin, Alcian blue (Alcian blue 8GX, Cyagen, S0152, China) and Oil Red O (Oil Red O stain kit, Solarbio, G1262,China) for half an hour, the sections were then examined under light microscopy (DMRXA2, Leica Microsystems Wetzlar GmbH, Wetzlar, Germany). To compare the healing effects of the injured tendon between the two groups, we used an established histological scoring system [18]. The score of the intact group was defined as 18 points. The evaluation indicators include A: ECM organization of the whole tendon(2 points), B: proteoglycan content (Alcian blue staining) (1 point), C: cellularity/cell-matrix-ratio(2 points), D: cell distribution(1 point), E: cell nucleus morphology(2 points), F: organization of repair tissue of the tendon callus(2 points), G: transition from defect to normal tissue(2 points), H: configuration of callus(1 point), I: degenerative changes/tissue metaplasia(3 points), J: vascularization in the defect area(1 points) and K: inflammation(1 point). Five images from each group at weeks 2 and 4 were randomly selected to be analyzed. Histological analysis was performed using the double-blind method and was completed by two independent assessors (Jingjing Wang and Kang Lu).

### Immunofluorescence staining

Firstly, the sections were rewarmed in room temperature for 30 min, then washed three times with PBS and blocked with 2% goat serum. The sections were then washed with PBS for 5 min three times and later incubated with the primary antibodies (anti-FABP4 (1:1000), Abcam (ab92501)) at room temperature for 12 h. After washed with PBS three times, the sections were incubated with the secondary antibodies (Santa Cruz Biotechnology (sc-2359), 1:200) for 30 min. Nuclei were counterstained with DAPI (Sigma Aldrich) for 15 min. The sections were observed and photographed with a laser scanning confocal microscope (Olympus IX70, Tokyo, Japan).

### Immunohistochemical staining

Sections were washed three times with PBS and then were blocked with 2% goat serum. The sections were incubated with primary antibodies (anti-Col-1(1:100), Abcam (ab92501), anti-Col-3(1:100),Abcam (ab7778), anti-MMP-1(1:100), Bioss (bs-4597R), and anti-TIMP-1(1:100), Bioss (bs-0415R)) at room temperature for 12 h. After washing with PBS three times, sections were incubated with secondary antibodies (Bioss,1:200) for 20 min and then were treated with Horseradish Peroxidase for 20 min. Sections were treated with DAB for approximately 1 min under microscope observation. After the samples were washed in water for 10 min the sections were treated with hematoxylin for approximately 30 s and were then washed slightly in water for 10 min and heated in 60 °C water for 15 min. Finally, the sections were dehydrated and sealed.

### Micro-CT scanning

Ectopic ossification at the tendon defect was observed using micro-CT with the same method we used before [19]. Patellar tendons that were to be analyzed were collected at 8 weeks post injury (*n* = 6), and then were wrapped in a homemade plastic tube and placed in the sample rack of a micro-CT system (Bruker MicroCT, Kontich, Belgium). The parameters were set as: voltage, 60 kV; current, 166 μA; and effective pixel size, 10.699940 μm. Using Skyscan (BRUKER, Germany) software, we set the threshold gray value to 50. The system automatically analyzed the volume of substances that had gray values greater than 50, which was the volume of the ectopic ossification blocks we observed.

### Real-time quantitative PCR

All the procedures were conducted as previously described [20]. Briefly, the mRNA expression levels of tendon-related (collagen 1a, Tenomodulin, Scleraxis), chondrocyte-related (collagen 2a, aggrecan and Sox9) and adipocyte-related (PPARγ, C/EBPα and FABP4) genes were determined using RT-PCR. Total RNA was extracted from tendons using TRIzol reagent (Takara, Dalian, China). cDNA was synthesized from total RNA using a Superscript III first-strand synthesis kit (TaKaRa). RT-PCR was performed using a SYBR Green RT-PCR kit (TaKaRa) and an ABI Prism 7900 Sequence Detection System (PE Applied Biosystems, Foster City, CA, USA). Expression levels were calculated relative to the expression of the housekeeping gene glyceraldehyde 3-phosphate dehydrogenase (GAPDH) with Bio-Rad CFX-Manager (Bio-Rad, USA). The relative expression levels of genes were calculated using the 2^-∆∆Ct^ method. The primer sequences used in these experiments are listed in Table 1.

**Table 1:** Primers sequence for PCR analysis

Primer	Sequence
Col1a	Forward 5′-TGGTGAGACGTGGAAACCTG-3’
	Reverse, 5′-CTTGGGTCCCTCGACTCCTA-3’
TNMD	Forward 5′-GCGACAATGTGACTATGTAC-3’
	Reverse, 5′-GTCTTCTCCACCTTCACTTG-3’
Scx	Forward 5′-AGAGACGGCGGCGAGAAC-3’
	Reverse, 5′-AATCGCCGTCTTTCTGTCACG-3’
TIMP3	Forward 5′-GGATGTGGAGCTGAAGGA-3’
	Reverse, 5′-GATGTGAGGCAGGGAAGA-3’
MMP3	Forward 5′-GGGCTATCCGAGGTCATGAAG-3’
	Reverse, 5′-CTTTGTGCCAATGCCTGGAA-3’
FMOD	Forward 5′-AAGATCCCTCCCGTCAACAC-3’
	Reverse, 5′-GCTTGATCTCGTTCCCATCCA-3’
PPARγ	Forward 5′-CCTTTACCACGGTTGATTTCTC-3’
	Reverse, 5′-GGCTCTACTTTGATCGCACTTT-3’
C/EBPα	Forward 5′-TACCTGGGCTACCAGGCGA-3’
	Reverse, 5′-CGCGCCGCATCTTGTACTC-3’
FABP4	Forward 5′-CGAGATTTCCTTCAAACTGGG-3’
	Reverse, 5′-TCTTGTAGAAGTCACGCCTTTC-3’
Aggrecan	Forward 5′-GGCGTCCAAACCAACCCGA-3’
	Reverse, 5′-GGAGCTGATCTCATAGCGATC-3’
Sox9	Forward 5′-GAGAACGCACATCAAGACGGA-3’
	Reverse, 5′-TGTAGGTGAAGGTGGAGTAGAG-3’
Col2a	Forward 5′-ATGCAGTCTTTCTTCGGCTTAG-3’
	Reverse, 5′-TCATCTGGACGRRAGCGGTGTT-3’
GAPDH	Forward 5′-TGACTTCAACAGCAACTC-3’
	Reverse, 5′-TGTAGCCATATTCATTGTCA-3’

### Western blotting

After collection, the patellar tendons were washed twice with PBS, and the tendons were cut as much as possible after weighing. Then, the lysis buffer (Thermo Fisher Scientific Inc., Rockford, IL) was added to the 300 mg of tissue in a proportion of 1.2 ml. After lysis on ice for 30 min, the lysis buffer was centrifuged at a speed of 13,500 rpm for 15 min. To the supernatant was added 25 μl of 5X loading buffer per 100 μl supernatant, and the mixture was boiled for 5 min. Total protein concentrations were determined by a bicinchoninic acid (BCA) protein assay kit (Thermo Fisher Scientific Inc). Equal amounts of tissue lysates (30 μg/lane) were subjected to sodium dodecyl sulfate polyacrylamide gel electrophoresis (SDS-PAGE) and were then transferred to polyvinylidene difluoride membranes. After being blocked by 5% non-fat milk containing 0.1% Tris buffered saline with Tween (TBST) at room temperature for 2 h, the membranes were incubated overnight at 4 °C with primary antibodies including anti-Col-1 (1:500; Abcam (ab34710)), anti-Col-3 (1:500; Abcam (ab7778)), anti-TNMD (1:1000; Abcam), anti-Biglycan(1:500; Proteintech(16409)), anti-matrix metalloproteinase (MMP-3 (1:500; Cell Signaling Technology), anti-TIMP-3 ((1:500); Proteintech (10858)), anti-Col-2 (Abcam (ab34712)), anti-aggrecan (Abcam (ab36861)), anti-Sox9 (Cell Signaling Technology(82630S)), anti-FABP4 (Abcam (ab92501)), and anti-glyceraldehyde-3-phosphate dehydrogenase (GAPDH) (Proteintech (HRP-60004)). After being washed three times in 0.1% PBST, membranes were incubated with a goat anti-rabbit IgG (H&L)-horseradish peroxidase conjugate (Proteintech) at room temperature for 2 h. Protein bands were detected by a chemiluminescence detection kit (LI-COR Biosciences, Lincoln, NE).

### Statistical analysis

All measurement values are presented as the mean values±standard deviations. A Student’s t-test or Mann–Whitney test was used to identify significant differences. A *p* < 0.05 was considered to indicate a statistically significant difference between groups.

## Results

### Histological scores showed that the healing effect in aged rats was worse than it was in young rats

**Fig. 1:**
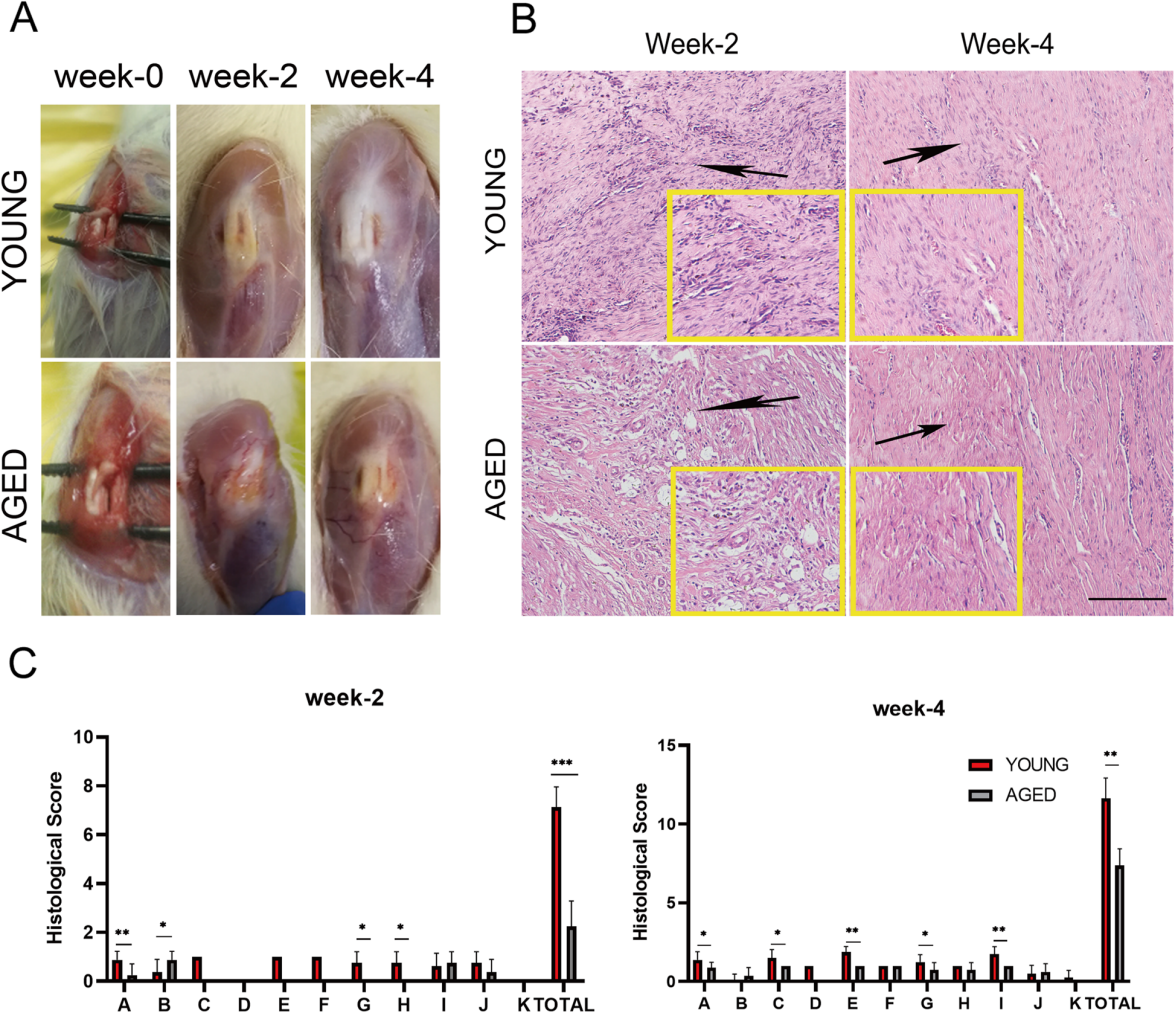
The comparison of the gross appearance, hematoxylin and eosin (H&E) staining and histologic scores of the patellar tendon defects in young and aged rats. (**A**). The representative images of gross appearance of the patellar tendon at weeks 0, 2 and 4 postoperatively. (**B**). The representative images of patellar tendons of young and aged rats were observed by HE staining. The scale bar is 200 μm. (**C**). Histologic scores of young and aged rat patellar tendons. A: ECM organization of the whole tendon, B: proteoglycan content, C: cellularity/cell-matrix-ratio, D: cell distribution, E: cell nucleus morphology, F: organization of repair tissue of the tendon callus, G: transition from defect to normal tissue, H: configuration of callus, I: degenerative changes/tissue metaplasia, J: vascularization in the defect area and K: inflammation. *:*p* < 0.05, **:*p* < 0.01. *n* = 5

To evaluate the influence of aging on tendon healing, we compared the effects of patellar tendon healing between young and aged rats using a histological scoring system established by previous researchers. There seemed to be more scar-like tissues on the surface of tendon defects in aged rats than there were in young rats (Fig. 1A). HE staining showed that the arrangement of collagen fibers in the young group was more orderly than that in the aged rats (Fig. 1B). The score of the young group was 7 ± 0.816 at week 2, while that of the aged group was 2.5 ± 1.291. At week 4, the score of the young group was 11.25 ± 1.258 and that of the aged group was 7.25 ± 0.957. In detail, the subgroups A, B, G, and H at week 2 and subgroups A, C, E, G, I at week 4 showed significant differences between young and aged rats. There are significant differences between the two groups’ total scores both at 2 (*p* = 0.001) and 4 (*p* = 0.002) weeks postoperatively (Fig. 1C).

### Collagen synthesis decreased in aged tendons

**Fig. 2:**
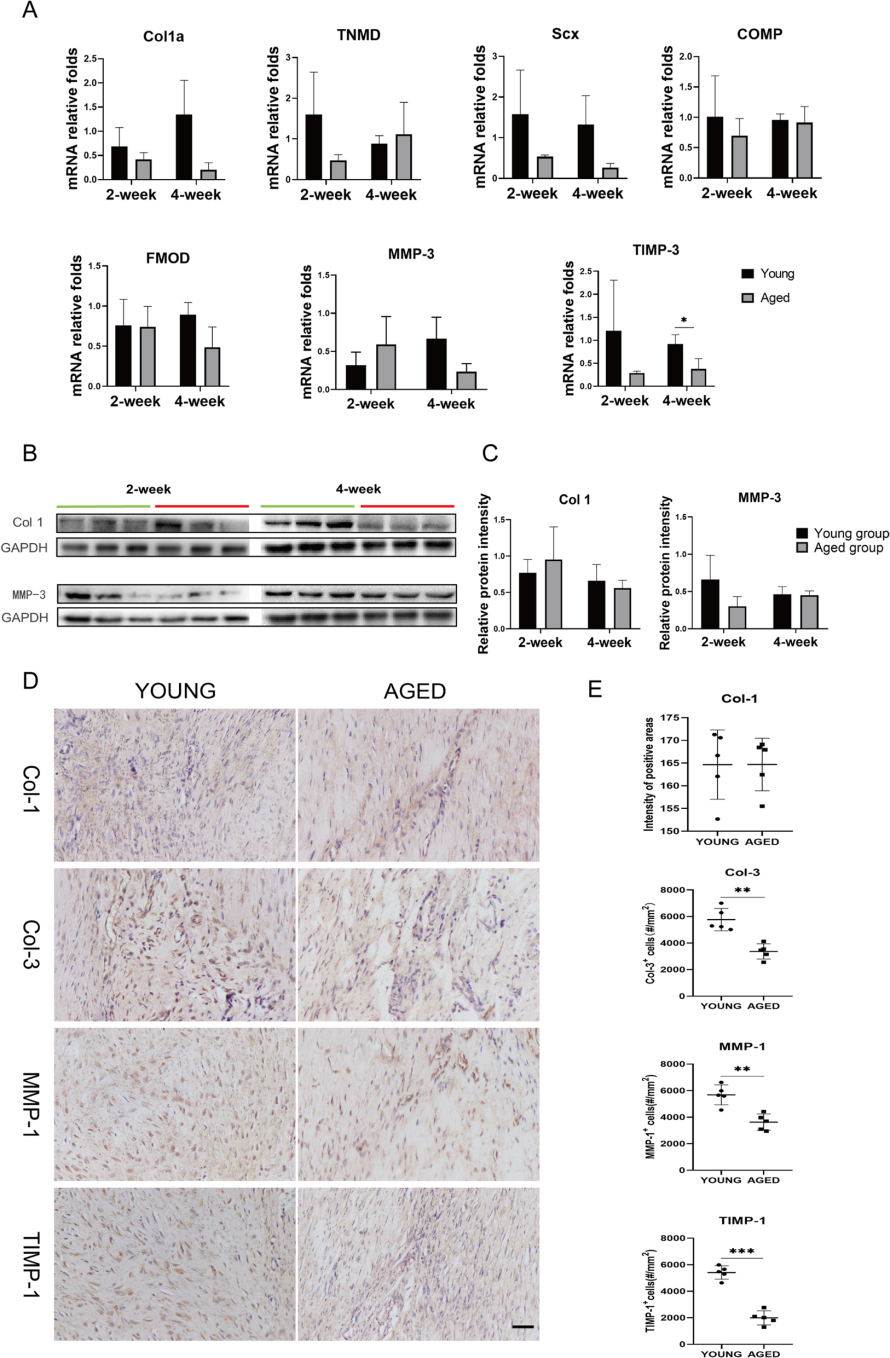
Biochemical analysis of tenogenic and repair-related markers in patellar tendons from young and aged rats. (**A**). Quantitative reverse-transcription polymerase chain reaction was used to determine the gene expression levels of Col1a, TNMD, Scx, COMP, FMOD, MMP3 and TIMP3 in young and aged rat tendons at weeks 2 and 4. Glyceraldehyde 3-phosphate dehydrogenase (GAPDH) was used as an internal control. (**B**). Western blot analysis of the protein expression levels of Col-1 and MMP-3. GAPDH was used as an internal control. (**C**). Quantitative analysis of western blot results. *p < 0.05, **p < 0.01, ***p < 0.001, and *n* = 3. (**D**). Comparison of the expression levels of Col-1, Col-3, MMP-1 and TIMP-1 in young and aged rat tendons by immunohistochemical staining. Black scale bar: 20 um. (**E**). Quantitative analysis of the immunohistochemical staining of Col-1, Col-3, MMP-1 and TIMP-1. *p < 0.05, **p < 0.01, ***p < 0.001, and n = 5

To determine the molecular mechanism of aging influencing healing between the two groups, biochemistry experiments were conducted. The RT-PCR results in both groups showed no significant difference in the main protein in tendon Col-1 and the tenogenic and repair-related markers tenomodulin (TNMD), Scleraxis (Scx), Cartilage oligomeric matrix protein (COMP), fibromodulin (FMOD) and Matrix Metalloproteinases-3 (MMP-3) at weeks 2 and 4. The gene expression of TIMP-3 was decreased in the aged group at week 4(p = 0.036) (Fig. 2A). Though the results showed that there was no difference in the expression of Col-1 and MMP-3 at both weeks 2 and 4(Fig. 2B&C), immunohistochemical results demonstrated that the expression levels of Col-3(*p* = 0.001), MMP-1(p = 0.001) and TIMP-1(*p* = 0.000) between the two groups were significantly higher in young rat tendons at week 4(Fig. 2D&E) and there was no difference in the expression of Col-1.

### The chondrogenic-related marker aggrecan was decreased in the process of tendon healing in aged rats

**Fig. 3:**
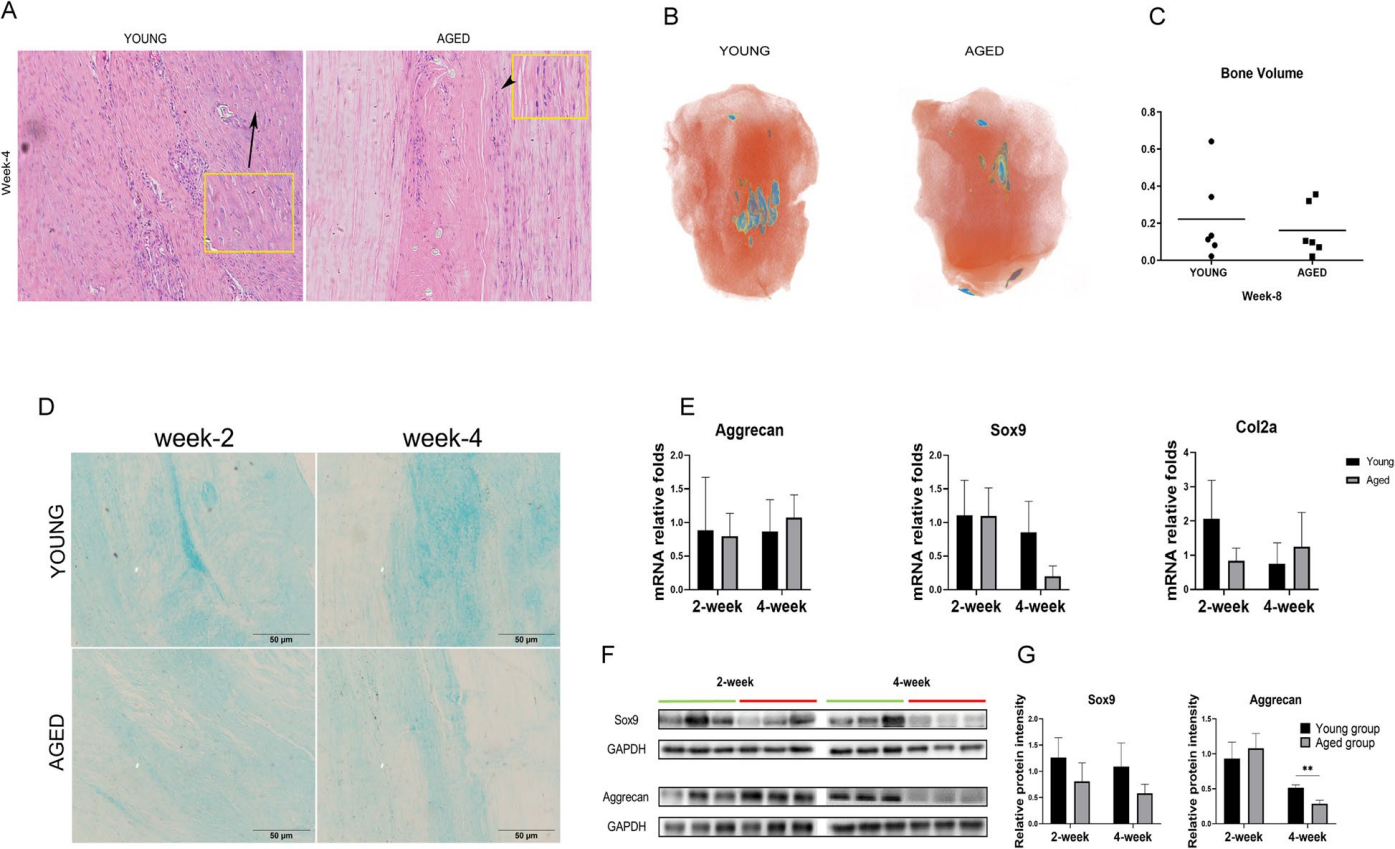
Analysis of the chondrogenic changes in the patellar tendons in young and aged rats. (**A**). Representative images of HE staining of the patellar tendon at week 4 in aged and young rats. Arrow head: chondrocyte-like cells in tendon. (**B**).Three-dimensional reconstruction of the patellar tendon. Red: patellar tendon; Blue: bone-like tissues. (**C**). Quantitative analysis of ectopic ossification of patellar tendons from young and aged rats 8 weeks post injury. (**D**). Representative images of Alcian blue staining of the patellar tendon at weeks 2 and 4 in young and aged rats. (**E**). RT-PCR of the gene expression levels of aggrecan, Sox9 and col-2. GAPDH was used as an internal control. *p < 0.05, **p < 0.01, ***p < 0.01, and n = 3. (**F**). Western blot analysis of the protein expression levels of aggrecan and Sox9. GAPDH was used as an internal control. (**G**). Quantitative analysis of western blot results (n = 3)

At week 4, we found the presence of cartilage or osteoid tissue in the tendon samples of both groups (Fig. 3A). Therefore, micro-CT was used to scan the patellar tendon 8 weeks after injury and found that there was a greater occurrence of osteoid tissue in the patellar tendon of the young rats than there was in the aged rats (Fig. 3B); however, there was no significant difference between the two groups (Fig. 3C*, n =* 6). Alcian blue staining was performed to measure the content of sulphated proteoglycans [21]. Alcian blue staining of the patellar tendon suggested that the sulphated proteoglycans in the patellar tendon of aged rats decreased relatively at weeks 2 and 4 (Fig. 3D). At the same time, we examined the chondrogenic markers aggrecan, sox9 and collagen II, and though we found no significant differences in gene expression, the expression of aggrecan at the protein level was much higher in young rats(*p* = 0.003) (Fig. 3E, F & G).

### Increased accumulation of adipocytes was found in aged rats during tendon healing

**Fig. 4:**
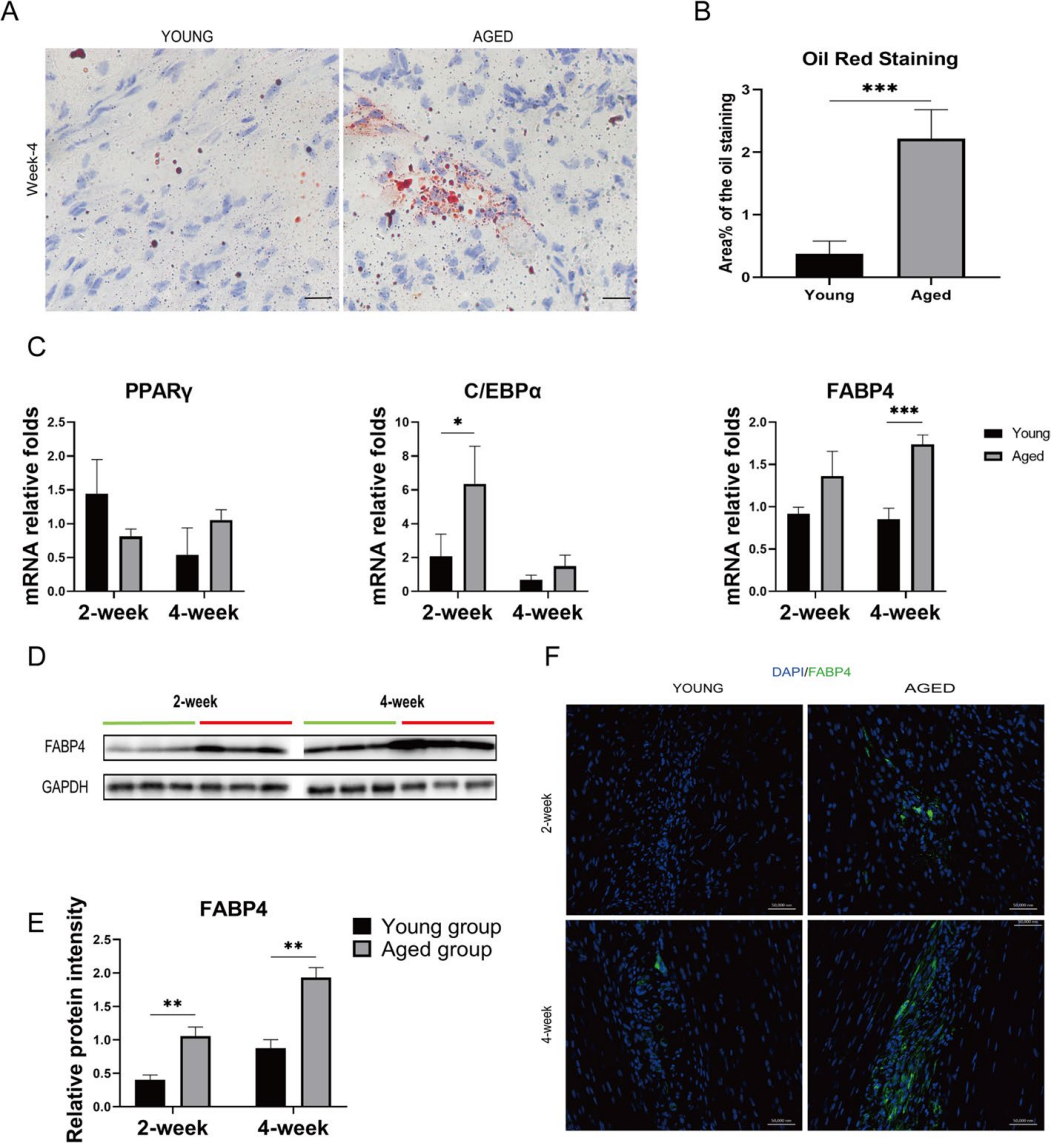
Analysis of adipogenic changes in the patellar tendons in young and aged rats. (**A**) Oil Red O Staining of the tendons of young and aged rats. (**B**). Quantitative analysis of area% of Oil Red O (*n* = 9). (**C**) Quantitative reverse-transcription polymerase chain reaction of the gene expression levels of PPARγ, C/EBPα and FABP4. (**D**). Western blot analysis of the protein expression level of FABP4 (n = 3). (**E**). Quantitative analysis of western blot results. (**F**). Immunofluorescence staining of the patellar tendons from young and aged rats

*A*dipocyte accumulation after tendon injury is very common and hampers the tendon injury healing process. To analyze adipocyte accumulation in the tendon, Oil Red O staining, PCR, western blot and immunofluorescence staining were performed. Oil Red O staining showed that there were more adipocytes(*p* = 0.000) in the aged group at weeks 4 post injury than there were in the young group (Fig. 4 A&B). PCR results indicated significant increases in C/EBPα at weeks 2 (*p* = 0.045) and FABP4 at week 4 (*p* = 0.001) (Fig. 4 C). Western-blot results showed significant increases at weeks 2 (*p* = 0.002) and 4 (p = 0.001) in the expression of FABP4 in the tendons of aged rats (Fig. 4D&E); the same result was further confirmed by immunofluorescence staining, which showed that FABP4 was highly expressed in regenerated tissues (Fig. 4F).

## Discussion

Tendon healing has always been a challenging problem, especially for aged people. The focus of studies on age-related tendon injury and tendinopathy is the influence of aging on tendon structures, compositions or functions, but such a focus ignores how aging affects healing responses. The healing mechanism of tendon injuries in aged rats remains unknown. It is of great importance to determine the key factors influencing tendon healing and the molecular mechanisms.

Perfect re-establishment of an injured tendon is supposed to recover well-organized collagen structure; both abnormal ectopic endochondral ossification and adipocyte accumulation can interrupt the natural healing process. In histological results, we found that the organization of the repair tissue of the tendon callus was completely changed in aged tendons. In addition, there seemed to be less inflammatory cells infiltrated in week 2 aged tendons, though there was no significant difference between groups. In this research, there were some difference between young and aged tendons in term of repair-related makers. We found the gene expression of TIMP-3 and the protein expression levels of Col-3, MMP-1 and TIMP-1 were significantly decreased in week 4 aged tendons. Col-3 can form heterotypic fibrils in combination with Col-1 and is mainly involved in tendon repair and growth [22]. The downregulation of Col-3 in aged rats demonstrated that the healing process may proceed more slowly than that in young rats. Matrix metalloproteinases are a large family of zinc-dependent endopeptidases that regulate ECM remodeling [23]. The function of matrix metalloproteinases is to modify all components of the ECM, including collagens, proteoglycans and fibronectins [24]. Collagen breakdown is triggered by MMP-1, and after degradation by MMP-1, other MMPs can complete collagen degradation. TIMP-1 is the TIMP that acts in the inhibition of MMP-1. TIMP-1 and TIMP-3 are thought to maintain tendon homeostasis with MMPs [25]. Furthermore, TIMP-3 is also involved in promoting cell proliferation and regulating angiogenesis and apoptosis [26]. The different expression levels of MMP-1, TIMP-1 and TIMP-3 may account for the poor organization of repair tissue in aged rats and by balancing the expression of MMPs and TIMPs tendon regeneration might be effectively improved.

In this study, we found that cartilage-like cells appeared around the tendon defect 4 weeks after the injury. In the histopathological studies of chronic tendinopathy, some chondrocyte-like cells were observed surrounding the microtear sites within the Achilles tendon in the early period of the disease [27, 28], and some severe late changes could also be seen, such as tendon calcification, cartilage and ossification [29]. Similar problems have been detected in human patients with Achilles tendinopathy [30]. The results from the Alcian blue staining for the patellar tendon hinted that the proteoglycan content was greater in young rats than in aged rats. In addition, the important chondrogenic-related marker aggrecan was simultaneously reduced. The results above indicated that there may be an erroneous presence of chondrocytes in both groups and there were more in the young group. However, micro-CT scan results of the tendons at week 8 showed ectopic ossification in both the aged and young rats, while the content in the tendons of aged rats appeared to be lower; nevertheless, further quantitative analysis showed no significant difference between the two groups.

Adipocyte accumulation is one of the most common pathological changes that occurs in tendinopathy or in ruptured tendons [31, 32]. The Oil Red O staining results are a direct indicator of lipid accumulation, and our results suggested that more adipocytes accumulated around the tendon defect 4 weeks after injury. Meanwhile, the qPCR, western blot and immunofluorescence staining results showed a higher expression of the adipogenic marker Fatty acid-binding protein 4 (FABP4), which indicated an abnormality in the healing process of aged rats. FABP4, also known as aP2, is a member of the intracellular FABP family, which binds long-chain fatty acids and other hydrophobic ligands [33, 34] and is involved in fatty acid uptake, transport, and metabolism [35, 36]. We believe that this may indirectly impair tendon repair and it is useful to limit the adipocyte accumulation. In our previous study on tendon healing, we found that the abnormal differentiation of TSCs is the key reason for adipocyte accumulation [17]. Furthermore, other studies have also found that there is greater fatty content in aged animals than in young animals [13, 37]. However, it is unclear whether the adipocytes that accumulated were a result of local stem cell differentiation or metastasis of the surrounding tissue. The mechanism of adipocyte accumulation in aged rats needs to be investigated in further studies.

There are still several limitations in this study. First, we did not expand the study to patients in a clinic, and the tendons were not of human origin. Second, we selected 2 and 4 weeks after injury as the time points of analysis; thus we may miss some important information at other time points. Last, we did not perform in vitro experiments to identify the changes that aging causes.

In many tissues, cell senescence is induced by aging [38, 39], which impairs wound healing [40]. In recent years, a small population of stem cells has been identified in tendons from several species [41, 42], and they have been termed tendon-derived stem/progenitor cells (TSPCs). TSPCs have characteristics of clonogenicity, self-renewal and multilineage differentiation, and they express key tendon-related genes, such as Scleraxis (Scx) and tenomodulin (Tnmd) [43]. Moreover, tendon-specific stem cells differentiate into tenocytes by default [44]. Studies have shown that TSPCs could promote tendon repair and regeneration [45]. Owing to these capabilities, TSCs play a crucial role in tendon maintenance and regeneration after injury [46, 47]. The function of TSPCs is an important factor in studying the repair of age-related tendon injuries. As previously reported, TSCs require a specific microenvironment, the so-called stem cell niche, which may influence their stemness or differentiation capability [47, 48]. Do changes in the environment of aged TSPCs also affect their functions? How does the function of TSPCs change, and is this change key to tendon repair disorders in aged individuals? These questions need to be addressed further.

## Conclusions

In conclusion, our findings indicate that tendon healing is impaired in aged rats, and it is characterized by a significantly lower histologic score, decreased collagen synthesis and more substantial adipocyte accumulation in the patellar tendon after repair. To improve collagen synthesis and to inhibit adipocyte accumulation may benew targets for improving age-related tendon injuries.

## Supplementary Information

**Additional file 1.**

## Data Availability

All datasets used and/or analyzed during the current study are available from the corresponding author on reasonable request.
