# Supplementary figures and images for: Effects of aging on the histology and biochemistry of rat tendon healing
Source: BMC Musculoskelet Disord. 2021 Nov 15;22:949. doi: 10.1186/s12891-021-04838-w (PMC8594129; doi:10.1186/s12891-021-04838-w)

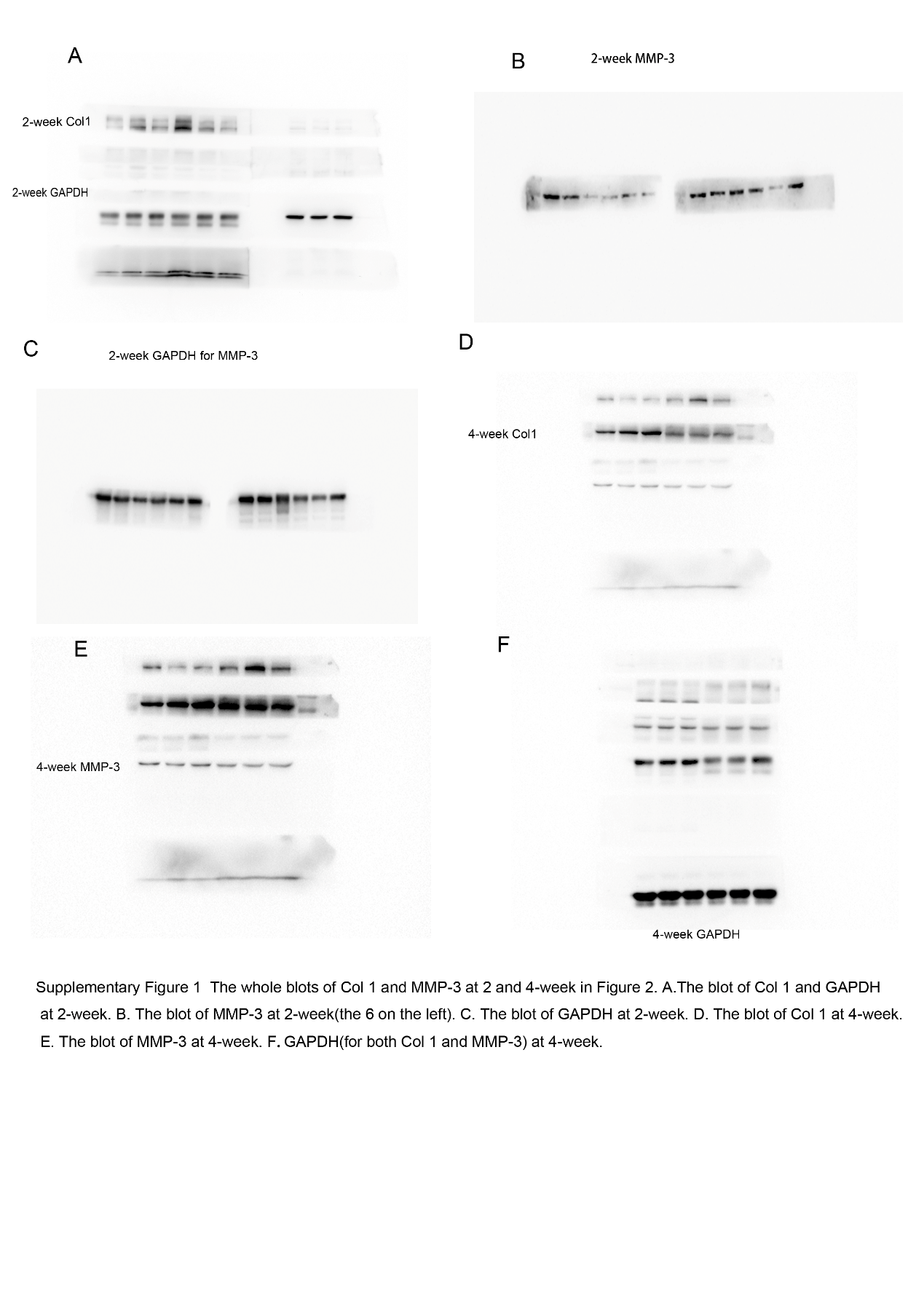

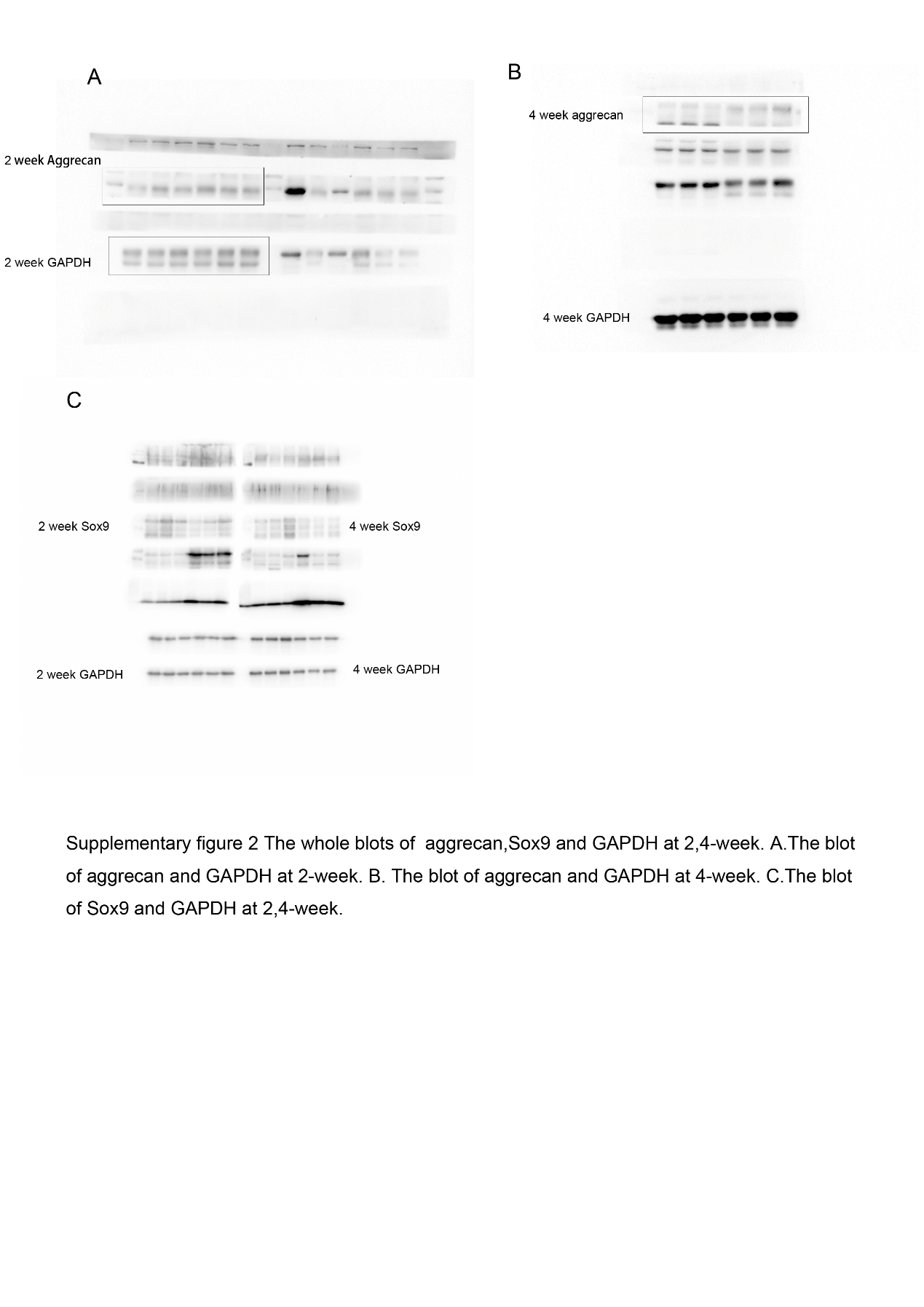

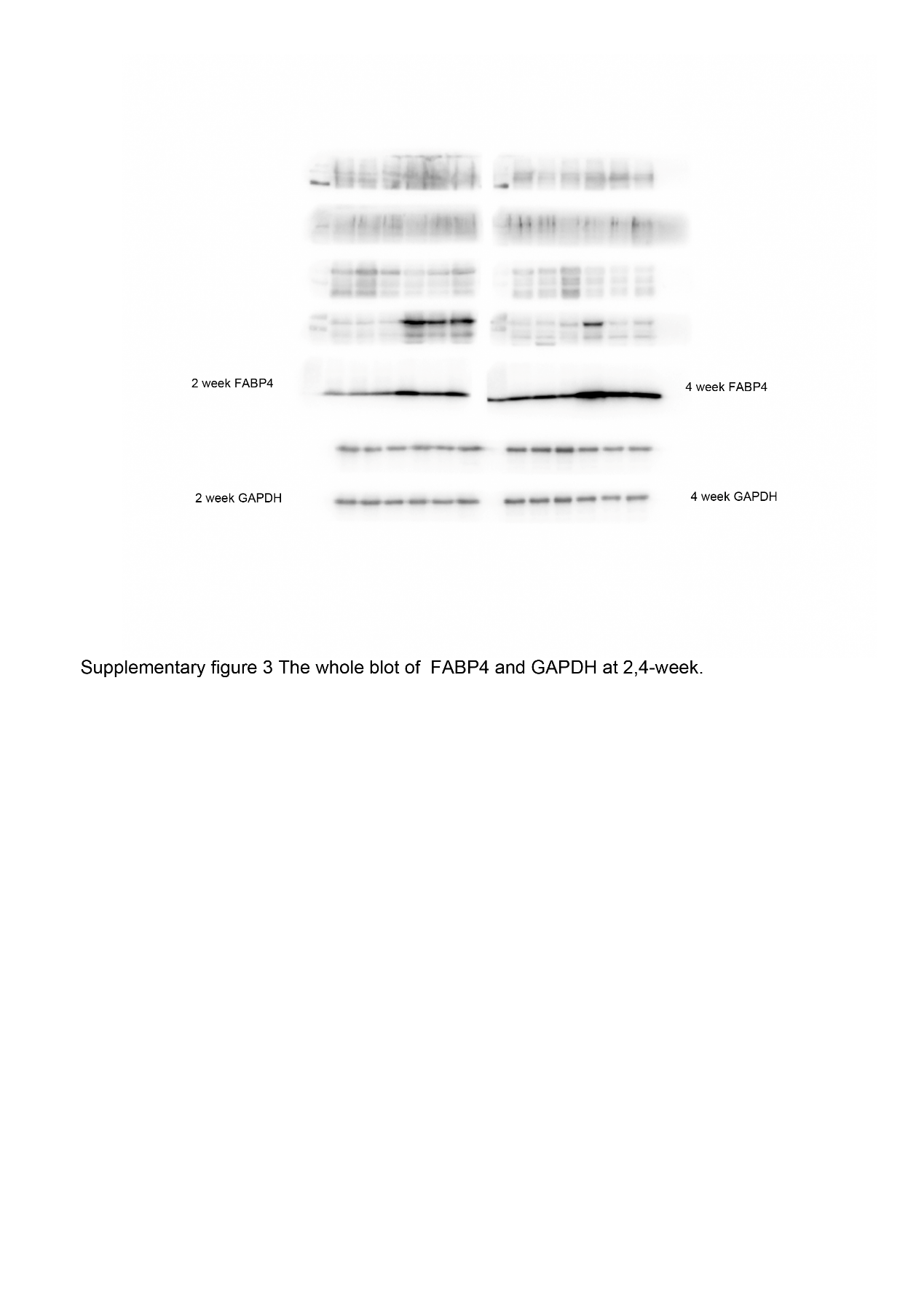

Supplement: Supplementary file 1 — Additional file 1. [file 12891_2021_4838_MOESM1_ESM.docx]
